# Zebrafish Embryos and Larvae as Alternative Animal Models for Toxicity Testing

**DOI:** 10.3390/ijms222413417

**Published:** 2021-12-14

**Authors:** Benedikt Bauer, Angela Mally, Daniel Liedtke

**Affiliations:** 1 Institute of Pharmacology and Toxicology, Julius-Maximilians-University, 97078 Würzburg, Germany; benedikt.bauer@uni-wuerzburg.de (B.B.); Mally@toxi.uni-wuerzburg.de (A.M.); 2 Institute of Human Genetics, Julius-Maximilians-University, 97074 Würzburg, Germany

**Keywords:** danio rerio, alternative methods, organ toxicity, 3R, transgenic animals

## Abstract

Prerequisite to any biological laboratory assay employing living animals is consideration about its necessity, feasibility, ethics and the potential harm caused during an experiment. The imperative of these thoughts has led to the formulation of the 3R-principle, which today is a pivotal scientific standard of animal experimentation worldwide. The rising amount of laboratory investigations utilizing living animals throughout the last decades, either for regulatory concerns or for basic science, demands the development of alternative methods in accordance with 3R to help reduce experiments in mammals. This demand has resulted in investigation of additional vertebrate species displaying favourable biological properties. One prominent species among these is the zebrafish (*Danio rerio*), as these small laboratory ray-finned fish are well established in science today and feature outstanding biological characteristics. In this review, we highlight the advantages and general prerequisites of zebrafish embryos and larvae before free-feeding stages for toxicological testing, with a particular focus on cardio-, neuro, hepato- and nephrotoxicity. Furthermore, we discuss toxicokinetics, current advances in utilizing zebrafish for organ toxicity testing and highlight how advanced laboratory methods (such as automation, advanced imaging and genetic techniques) can refine future toxicological studies in this species.

## 1. Introduction

The rising amount of experimentation utilizing animals throughout the last decades demands the active development of alternative methods and assays. Prerequisite to any biological assay utilizing animals is consideration about its necessity, feasibility, ethics and the potential harm caused. The imperative of these thoughts has led to the formulation of the 3R-principle [1], which today is well accepted as a scientific standard and which has been implemented in the legislation framework of animal experimentation worldwide. In recent years the necessity of adequate alternatives especially to mammals and higher vertebrates for regulatory safety testing has resulted in raised scientific interest to establish methods in a variety of species displaying biological properties more suitable for specific scientific investigations. One large group among these alternatives are ray-finned fish (*Actinopterygii*), comprising an enormous amount of approximately 30,000 different species including well-known laboratory fish species such as zebrafish.

### 1.1. Zebrafish—Species-Specific Advantages and Limitations

Today the predominantly used small fish model species in biomedical laboratories worldwide are zebrafish (*Danio rerio*) [2,3] and to a lesser extent killifish species, such as the Japanese Medaka (*Oryzias latipes*) [4,5]. Zebrafish are used as comparative model for a wide number of different basic research areas and as disease models, e.g., for cardiovascular disorders [6], bone research [7], immunology [8] and cancer [9]. The species shares similar to all other vertebrate species a basic chordate body plan: a vertebral column/notochord, a neurocranium/skull, neural crest cells, defined epidermal structures, sensory placodes, balance organ/labyrinth organ, and distinct neurological features. Several additional characteristics fostered the usage of these small laboratory fish, in particular: extracorporeal fertilization, fast embryonic development, relatively small body size, easy and cost-efficient animal handling, optical transparency of embryos and adult fish, established genetic and molecular laboratory protocols, as well as the potential to perform high-throughput assays [4,10,11]. The Zebrafish genome has been fully sequenced (current version: GRCz11), shares a high degree of genetic homology to higher vertebrates and displays a great number of common genetic features with other vertebrates [12,13] (https://www.ncbi.nlm.nih.gov/grc/zebrafish; accessed 9 December 2021). Besides advancing fundamental research, zebrafish studies have also provided valuable insights for the pharmaceutical industry, with several novel drugs targets originating from zebrafish work currently under investigation in human clinical trials, such as MEK inhibitors for lymphatic anomalies and potent melanoma inhibitors [14].

Besides these advantages and the genetic conservation, other biological aspects differ between fish species and mammals and are investigated by comparative physiology. First, fish have adopted to aquatic environments during evolution and have developed specialized anatomical features, e.g., gills, swim bladders, scales and extracorporeal fertilization. Although developmental similarities have been found in gills and in lungs, structural organization, developmental origin and physiological function remain rather different [15]. Secondly, loss of genes, neo-functionalization of gene products, and gene-duplication have accrued in a teleost-specific (and salmonid-specific) whole genome duplication during evolution [16,17]. These events resulted to some extent in gene expression changes, signalling pathway alterations and gene function adaptations. Therefore, special care has to be taken by direct comparison between fish species and higher vertebrate genomes, as evolutionary distance and several whole genome duplication events have to be considered and resulted in genetic diversity between species [17,18]. Third, fish have retained the capacity of regenerating organs after damage throughout their lifetime. Regenerating tissues include extremities, heart and neuronal cells and employ highly specialized molecular processes missing in higher vertebrates [19]. Besides these selected examples, a wider number of biological differences can be observed in organ development (e.g., sex differentiation), adaptive immunology, behaviour (e.g., parental care, social behaviour), and in neurology (e.g., lack of neocortex) [10]. Therefore, the transition of novel findings from fish directly to other common laboratory animals and humans is seldom straight forward and still needs validation in mammals. In accordance with these points, the suitability of a fish model to the specific scientific hypothesis and to the planned assay has to be carefully considered before conducting experiments in zebrafish. Nonetheless, by carefully taking in account these differences, a rising number of comparative interspecies studies has been successfully performed and the results are the foundation for implementation of fish species in investigation of molecular processes common to all vertebrates as well as their application in toxicological testing [20].

### 1.2. Prerequisites for Use of Zebrafish for Toxicity Testing

Fish species are widely used in ecotoxicology, e.g., by investigation of the impact of chemicals and environmental contaminants on fish populations [21,22]. Several fish species, including zebrafish, are integrated in the internationally accepted OECD Guidelines to assess systemic toxicity in fish, i.e., *The Testing of Chemicals with the Fish Acute Toxicity Test* (OECD 203) and *The Fish Embryo Acute Toxicity Test* (OECD 236) [23,24]. Currently the European Commission Directive 2010/63/EU, permits experimentation in fish embryos at earliest life stages without being regulated as animal experiments (Current form: http://data.europa.eu/eli/dir/2010/63/2019-06-26; accessed 9 December 2021 EFSA opinion: https://doi.org/10.2903/j.efsa.2005.292; accessed 9 December 2021). This includes zebrafish embryos and early larval stages until free-swimming and independent feeding, corresponding to 5 dpf (days post fertilization) after raising at 28.5 °C. These regulations thus allow toxicological studies in zebrafish at these early developmental stages as an alternative model to animal testing in other vertebrates, e.g., rodents, but often limits these investigations to developmental and to acute toxic effects. 

Similar to other animal experiments in toxicology, 3R should be strictly implemented in experiments using small fish species at all developmental stages. First, replacement methods, which avoid or replace the use of animals in research, such as cell culture systems, 3D tissue models, or organoid cultures [25,26,27] should be considered. However, for some applications in vitro models provide no adequate replacement, as systemic toxic effects, e.g., whole animal development or organ function, can best be investigated in living organisms [28,29,30]. Here, zebrafish embryos and larvae have been suggested as a second line of screening for hit to lead identification and optimization of new drug candidates in preclinical toxicity testing, following the first line of screening in cell culture-based high-through-put assays [31]. Only the top three candidate compounds, remaining from embryonal or larval zebrafish tests, are suggested to be investigated in traditional mammalian model systems, thereby reducing the numbers of used animals. Second, reduction methods that enable researchers to obtain comparable levels of information from fewer animals, or to obtain more information from the same number of animals should be used. The methods result in reduction of animal number but require rigorous strategic planning and standardization of experiments to minimize experimental variation. Examples relevant to tests in fish that help to reduce the number of animals per experiment are non-invasive imaging [32], intravital time-laps investigations [33] and proper selection/combination of fluorescent transgenic animals [34]. Third, refinement methods have to be considered, that alleviate or minimize potential pain, suffering or distress, and enhance animal welfare for the animals used. General pain scoring methods and analgesics in zebrafish are sparse nowadays, but are currently under development [35]. 

## 2. Consideration of Toxicokinetics

Embryonal and larval zebrafish offer great benefits for the identification of hazardous compounds. For human health risk assessment, however, translation of doses and concentrations employed in zebrafish to human equivalent doses is eminent and requires detailed knowledge of the toxicokinetics of the compound under investigation. Therefore, in the following two paragraphs we give a non-exhaustive overview of some of the characteristics and challenges of toxicokinetics in zebrafish embryos and larvae. 

### 2.1. Absorption and Distribution 

**Figure 1:**
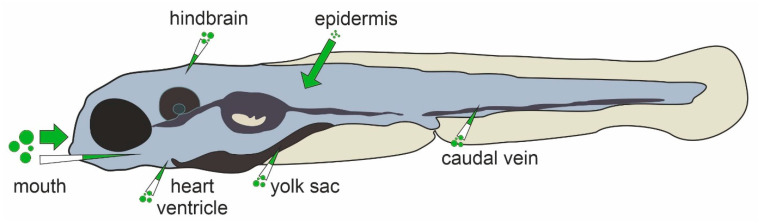
Possible compound application routes for larval zebrafish. Uptake routes which predominate during exposure by immersion are indicated by arrows, sites for compound microinjection by injection needles.

In humans and other higher vertebrates, compounds must pass physiological barriers, such as the epidermis, epithelial layers of the gastro-intestinal tract and the blood-brain barrier. In addition to these, zebrafish embryos are surrounded by the chorion, an acellular fetal envelope of 1.5–2.5 µm thickness. The chorion shields the zebrafish embryo until hatching at around 72 hpf (hours post fertilization) and contains pores with a diameter of 0.5–0.7 µm, preventing compounds larger than 3 kDa to freely pass [36]. However, the barrier function of the chorion, which varies between stages of embryonal development, may differ between compounds and exposure durations [36,37,38]. Thus, while chorion removal facilitates compound uptake, it is not obligatory for every compound. The epidermis is another factor that greatly influences compound uptake. While small diatomic molecules such as oxygen can easily pass the epidermal layer even in larval stages [39], large compounds may not be able to penetrate the epidermis [40]. Beginning at 60 hpf when the mouth starts to open, oral uptake gains increasing importance as a route of exposure to xenobiotics [3]. For both oral and epidermal exposure, immersion is the most common treatment method for zebrafish embryos and larvae due to the ease of application analogous to cell culture. It is important to consider that foreign compounds may be differentially absorbed by the embryonal and larval body, potentially resulting in low internal doses and correspondingly false negative findings [41,42]. Bioanalysis by LC-MS/MS of whole-body homogenates [43] or nanoscale blood samples [44] are therefore critical to verify internal exposure. Microinjection of compounds into the cardiac ventricle, caudal vein, hindbrain, yolk sac or into the intestinal lumen for microgavage may be used to overcome poor absorption [45,46]. Common routes of compound application in zebrafish larvae are summarized in Figure 1. Once absorbed, compounds are distributed throughout the embryonal and larval body. In zebrafish, chemicals have been shown to accumulate in different compartments. For instance, the melanin of the zebrafish eye, has been suggested as a binding site for basic drugs [47], consistent with findings from mammalian studies. Importantly, the yolk functions as a major compound depository, resulting in an overestimation of internal doses in the larval body [37,48]. As organs mature and the yolk is consumed over time, sites of compound accumulation can differ with increasing age from those at earlier developmental stages [49]. Furthermore, compound accumulation depends on the method of application. Methods suitable for studying the distribution of compounds into the different body regions of zebrafish include fluorescent dyes, radio-scintillation [50], and—more recently, MALDI-MS Imaging [51].

### 2.2. Metabolism and Excretion

Zebrafish express drug metabolising enzymes, including phase I enzymes such as Cytochromes P450 (CYPs), as well as sulfo- (SULTs) and UDP-glucuronosyltransferases (UGTs) which are involved in phase II xenobiotic metabolism [52,53,54]. CYPs are evolutionary conserved and show many orthologs between humans and zebrafish [55,56]. However, genetic synteny between a human CYP gene and its zebrafish ortholog does not necessarily lead to metabolism of the same substrate and, vice versa, absence of an ortholog can be substituted by other zebrafish CYP enzymes [57]. In addition, zebrafish CYP’s exhibit spatiotemporal differences in their expression profiles, with a strong increase in CYP gene expression after hatching [57,58,59]. Despite these potential limitations, zebrafish CYP orthologs frequently produce metabolites corresponding to those identified in mammals, as extensively reviewed by Anselmo de Souza et al. [54]. Richter et al. recently developed a larval zebrafish in vitro model for forensic toxicology that correctly predicted the human metabolites of a new synthetic cannabinoid [60]. Likewise, in the first few hours of development zebrafish start to express metabolic enzymes such as glutathione-S-transferases (GST’s), whose detoxification capacity in the mercapturic acid pathway was demonstrated recently in embryos and larvae exposed to the model GST substrate 2,4-dinitrochlorobenzene [61,62,63]. While elimination of xenobiotics from the human body takes place by clearance via the kidneys, bile/faeces and the lungs, in zebrafish, bile production and gills, which serves as a major respiratory organ, are not fully functional during the first 4 and 14 days of development, respectively [39,64]. Therefore, renal excretion is speculated to be the predominant route of elimination of xenobiotics in zebrafish larvae.

## 3. Application of Zebrafish to Assessment of Target Organ Toxicity

Zebrafish embryos and larvae have in the past been successfully used to investigate a range of different compounds, drugs or chemicals and to analyse their adverse effects in various target tissues. In this section we highlight application of zebrafish as a model for organ toxicity testing, with focus on embryonic and larval developmental stages up to 5 dpf, which fall under the European in vitro legislature. The studies presented in the following paragraphs (Table 1) focus on cardio-, neuro-, hepato- and nephrotoxicity as these toxicities are among the most common toxicities observed during human clinical trials and are subsequently responsible for the withdrawal of many drugs [65]. Further information about embryonal and larval zebrafish as a model for ocular, intestinal or endocrine toxicity can be found in the literature [20,66,67]. In assessing the significance of the organ toxicities reported in the studies described in the following paragraphs, the period of exposure needs to be critically considered. Even after 3 dpf, when most organs are well developed and zebrafish enter the free-swimming larval stage, zebrafish larvae still undergo developmental processes which might blur the line between developmental and acute toxicity. While typical manifestations of acute developmental toxicity are altered growth, systemic functional deficiencies (e.g., cardiovascular malformations), structural abnormalities, malformations and high death rates, investigation of organ-specific toxicity requires close consideration of drug application beginning, windows of exposure, investigated developmental stages, dose selection and inclusion of extensive controls to avoid masking of organ-specific adverse outcomes by developmental toxicity. 

### 3.1. Cardiotoxicity 

The two-chambered embryonic zebrafish heart comprises four distinguishable structures: atrium, ventricle, sinus venosus and bulbus arteriosus [79,80]. It starts beating at 20 hpf [79]. At 24 hpf the heart tube is completed and the division into two chambers occurs at 48 hpf [81]. Valves, however, are not present at 48 hpf but develop later until 5 dpf [80]. Because of this, regurgitation of the blood flow is possible during early life stages [82]. Despite the early onset of heart function, blood circulation is not essential until 7 dpf, when the larvae’s need for oxygen can no longer be covered solely by dermal diffusion [39]. This allows investigation of severe cardiac phenotypes in zebrafish, which in rodent embryos would most likely be lethal due to lack of oxygen supply by circulatory dysfunction [83]. Because of this, zebrafish embryos´ and larvae´s potential to model ischemic cardiac events during embryonic stages might be principally limited. Despite this, measurements in adult zebrafish showed that the zebrafish electrocardiogram (ECG) is more similar to the human ECG than that of rats and mice [70,84]. Further electrophysiological similarities were found in different zebrafish mutants, e.g., with the discovery of *zerg*, a zebrafish ortholog of the *hERG* channel [85,86], which is an important target in preclinical cardiotoxicity testing [87]. Due to this interspecies genetic and functional homology, impairment of heart function and morphology can be reproduced in embryonic zebrafish [88] after exposure to compounds known for their cardiotoxic effects in human clinical trials [89,90]. Examples of these compounds, recently shown to be cardiotoxic in embryonic zebrafish, include kinase inhibitors intended for chemotherapy [91,92], the antiarrhythmic drug verapamil [93] and the antihistamine terfenadine [94], that has been withdrawn from the market due to potentially lethal ventricular arrhythmia caused by prolongation of the QT interval [95]. Several studies (Table 1) support the model´s good sensitivity for cardiotoxicity, ranging between 85% and 100% [68,69,70,71]. 

The zebrafish heart features the advantage of being visible and optically transparent throughout embryonic and early larval stages. Thus, a plethora of non-invasive imaging assays, ranging from simple manual counting to large-scale automated imaging pipelines with corresponding software for evaluation, enable measurement of the embryonic zebrafish´s heartbeat [69,96,97,98,99,100]. However, depending on the setup, anaesthesia for positioning can pose a problem, as the commonly used anaesthetic tricaine (TMS, MS-222, Finquel, (3-aminobenzoic acidethyl ester methanesulfonate)) itself alters the embryo´s heart function [69]. Alternative anaesthetics, e.g., 2-phenoxyethanol, lidocaine and ketamine hydrochloride, are currently under debate and might be permitted for use on living zebrafish for heartbeat measurements. The shape and size of the embryonic zebrafish heart can be judged by simple light microscopy or by the use of transgenic lines like *myl7:GFP* (formerly known as *cmlc2:GFP*), which exhibits fluorescent cardiomyocytes [69]. Other parts of the vasculature can be visualized by transgenic lines marking endothelial cells, e.g., *fli1:eGFP* [101], while vascular blood flow can be measured by monitoring the fluorescent erythrocytes of the *gata1:dsRed* line [102]. Some commonly used transgenic lines for cardio-, neuro-, hepato- and nephrotoxicity are listed in Table 2. 

### 3.2. Neurotoxicity 

The gross organisation of the peripheral and central nervous system, as well as its neurochemistry, is conserved between zebrafish and mammals [115,116]. Differences consist in a general absence of a neocortex and stellate astrocytes, plus different development of the telencephalon in teleosts [117,118]. The zebrafish blood-brain barrier starts functioning at 3 dpf and prevents high molecular weight compounds from entering the CNS [119,120] but is not yet fully developed until 10 dpf [121]. Loosely myelinated axons are present at 3 dpf [122]. With progressing age of the larva, myelin tightens, and its amount increases [122]. Classical human (developmental) neurotoxins [123] such as the heavy metals lead [124] and mercury [125], as well as retinoic acid [126] and organophosphates [127] were also shown to alter the behaviour of embryonic and larval zebrafish. Larval behaviour can further be modulated by psychoactive compounds [115,128,129,130]. Large-scale studies, using the National Toxicology Program (NTP) 91-compound library (Table 1), revealed a sensitivity for detecting (developmental) neurotoxins above 66%, and up to 95%, when taking bioavailability into account [42,72,131]. 

Zebrafish embryos and larvae exhibit a rich repertoire of distinct behavioural patterns [132,133]. At early developmental stages, startle responses to different stimuli emerge, for instance to touch [134,135], abrupt changes of light, [136,137] and noise [138]. Spontaneous contractions of embryonic tails at 17–19 hpf [3,129,139] and thigmotaxis (“wall-hugging behaviour”) of 5 dpf larvae [140,141] are also features of the earliest zebrafish behavioural repertoire. These behavioural patterns are used as neurobiological endpoints in the assessment of developmental neurotoxicity in zebrafish embryos and larvae [142]. Commercially available tracking systems, e.g., Danio Scope (Noldus), MWP system (Zantiks) or ZebraLab (ViewPoint), allow the quantification of those endpoints in cell culture multi-well plates in a high-throughput fashion [143]. However, behavioural assays with zebrafish embryos and larvae are easily influenced by methodological factors, e.g., choice of zebrafish strain [144,145,146], light/dark condition during housing [42], vehicle concentration (e.g., DMSO) [146], or enzymatic dechorionation [131,147], thereby increasing outcome variance and compromising reproducibility. 

### 3.3. Hepatotoxicity 

The anatomy of zebrafish liver varies from mammals in several aspects. In contrast to the hexagonal lobules consisting of hepatocyte plates in mammals, zebrafish liver cells are arranged in tubules. Portal fields and metabolic zonation are missing, thus the familiar mammalian organizational divisions like liver acinus cannot be translated [73,148,149]. However, except for Kupffer cells, all hepatic mammalian cell types can also be found in zebrafish [64]. At 72 hpf, the zebrafish liver is perfused with blood [150] and is metabolically functional at 4 dpf [151], while biliary excretion can be measured starting from 5 dpf [64]. 

Phenotypes of liver toxicity in mammals include cholestasis, fibrosis/cirrhosis, inflammation and steatosis [152,153]. While these pathologic conditions are also observed in zebrafish larvae, as reviewed by Goessling et al. [154], not all of them have been shown to result from toxic insults. While compound-induced steatosis in zebrafish larvae has been observed in a number of studies [155,156,157], prominently with the focus on alcoholic fatty liver disease [158,159,160,161], cholestasis and inflammation have not yet been reported after toxin exposure [155,162,163,164]. Cholestasis occurs in mutant larvae [151] and as a drug-induced effect in adults [164]. Furthermore, alterations in bile acids regulation of the glucose and lipid metabolism pathway via Farnesoid X receptor (FXR) were detected in zebrafish larvae after hepatotoxin exposure with three model compounds for cholestasis, steatosis, and necrosis [165]. FXR is a nuclear receptor that is essential for de novo bile acid synthesis and is used as a drug target for the treatment of cholestatic conditions [166,167] However, xenobiotics do not seem to cause cholestasis in larval zebrafish, probably due to the late onset of bile production at 5 dpf [64,164]. The absence of inflammation is speculated to be related to the lack of Kupffer cells and the general underdevelopment of the adaptive immune system in zebrafish [168]. On the other hand, signs of fibrogenesis have been found by Zhang et al. who detected stellate cell activation and deposition of extracellular matrix proteins after exposure to ethanol [162,169].

Detection of hepatotoxicity in zebrafish larvae has often been carried out by the evaluation of morphologic endpoints. Transgenic fluorescent lines label hepatocytes, biliary and stellate cells (Table 2), thus allowing early quantification of reporter gene expression and liver morphology [107,170,171]. The latter can even be achieved with transparent wild type larvae, as changes in the liver can readily be imaged by light microscopy [155,172]. Steatosis can be visualized by whole-mount Oil Red staining [156,157,161,173]. Yolk retention serves as an indicator of hepatic function as it is utilized by the liver before onset of external feeding [172]. In a collaborative effort between Evotec, Pfizer and Johnson & Johnson, a combination of morphologic endpoints (scoring liver size changes, liver tissue degeneration and liver dysfunction at 120 hpf after 48 h incubation time) supported the added value of larval zebrafish in combination with cell-culture based high content screening assays for hepatotoxicity testing [73], therefore rendering embryonic zebrafish an attractive alternative in vivo model for liver toxicity. 

### 3.4. Nephrotoxicity 

Larval zebrafish possess a pronephros—the simplest form of a vertebrate kidney—which consists of two nephrons that share a single glomerulus [174]. As freshwater fish do not concentrate their urine [175], the zebrafish pronephros lacks the loop of Henle [176]. Additionally, zebrafish nephrons contain the so-called corpuscles of Stannius, which comprises endocrine glands and is responsible for calcium and phosphorus homeostasis [177]. Despite these differences, the zebrafish pronephros is highly homologous to humans. Investigations of the expression patterns of evolutionary conserved transporters (e.g., *slc20a1*, *slc4a4*) revealed a comparable nephron segmentation with different proximal and distal parts [178]. The endocytic transporter complex megalin/cubilin, that plays an important role in the reabsorption of proteins and compounds with peptide structure from the tubular lumen, is also expressed in a cell type-specific manner [179]. Pronephros organogenesis is finished at 3 dpf and the organ has gained its final shape that it will maintain until 12 dpf [112]. Glomerular filtration, however, already starts earlier at 48 hpf [180]. 

Several researchers have made use of the homology between zebrafish and mammalian kidney to study the impact of various toxins on renal development [78,181,182,183,184,185]. In these studies, the fluorescent reporter line *wt1b:GFP*, which marks the glomerulus and proximal tubules, has been widely used (Table 2). Using this transgenic line, Westhoff and colleagues developed an automated imaging pipeline (Table 1) in which adverse effects of nephrotoxic drugs on the developing embryonic kidney´s morphology was imaged in phenyl thiourea (PTU; 1-phenyl-2-thiourea) depigmented embryos [77,186]. Using this platform, morphologic alterations, including tubular distance, angle and degree of glomerular fusion were found to correlate well with histopathological findings observed using routine H&E staining [76]. As emphasized by the authors of the study, impairment of pronephros function is not necessarily associated with morphological alterations of the pronephros. 

For the assessment of the pronephric function, clearance experiments with injection of fluorescent low molecular weight dextrans into the circulatory system are commonly used [75,78,181,182,187,188,189,190]. As zebrafish rely solely on the kidney for excretion of substances until 14 dpf, when the gills are fully functional [39], the decrease in fluorescence is directly linked to renal clearance of the fluorescent low molecular weight dextrans. Hentschel et al. were the first to apply this technique to study effects of cisplatin and gentamicin on renal function in zebrafish. Disturbance of renal clearance was in concordance with histopathological findings in zebrafish exposed to cisplatin and gentamicin [187]. Glomerular slit diaphragm integrity can be tested by using dextrans with higher molecular weight [191]. Due to their larger size, the fluorescence-coupled sugars are only excreted if podocyte integrity is lost, which allows the assessment of glomerular proteinuria. This principle has been successfully used for assessing puromycin induced damage to the glomerular slit diaphragm [191,192]. A non-invasive method for the detection of inducible damage to the glomerular filtration membrane was developed by Zhou et al. who detected leakage of fluorescence-tagged vitamin D binding protein into the culture medium with a GFP-ELISA [193]. Possibly, this ELISA approach for the detection of fluorescence-labelled biomarkers could also be carried out with common urinary kidney injury biomarkers [194]. Recently, Bauer et al. reported upregulation of the nephrotoxicity biomarkers *hmox1*, *kim-1*, *ctgf*, *clu* and *spp1* after treatment of 3 dpf zebrafish larvae with nephrotoxins for 48 h, involving aristolochic acid, gentamicin, ochratoxin A and cadmium chloride [74]. Upregulation of these putative biomarkers in response to treatment with these model nephrotoxins was in concordance with histopathological alterations [74,195]. Similarly, 5 dpf larvae previously exposed by immersion to tenofovir, paracetamol and gentamicin for 24 h also displayed morphologic changes in the proximal convoluted tubule, including ultrastructural mitochondrial alterations reminiscent of effects observed in mammals [75]. 

## 4. Methodological Approaches to Toxicity Testing in Zebrafish 

There is increasing interest in toxicology, particularly in the field of systemic toxicity testing, to utilize the species-specific advantages of zebrafish to replace experiments in rodents. In this section we outline the benefits of transgenic zebrafish lines along with current challenges and potential pitfalls, including lack of standardized methods. 

### 4.1. Transgenic Zebrafish Lines and In Vivo Imaging 

One of the mayor advantages is the usability of zebrafish for a wide array of different, high-throughput amenable imaging techniques. Especially visualization of developmental processes during early development (≤5 dpf) in living embryos have greatly advanced knowledge of cellular process timing and spacing. Development of advanced imaging systems for zebrafish investigations; e.g., light-sheet, multi-photon or second-harmonic imaging microscopy [32] enable continuous investigation of three-dimensional processes in real-time without interference of molecular and physiological processes [196,197]. Moreover, the small embryo size enables automated imaging systems to investigate chemical compounds on embryonic development and can be further enhanced by combination with fluorescent tissue markers [77]. Development of novel imaging techniques, enabling detailed, non-invasive visualization of adult organs is ongoing and is expected to result in methods suitable for assessment of subacute to chronic toxicological effects [198]. 

When it comes to imaging fluorescent zebrafish lines, pigmentation can pose a problem, depending on the localization of the reporter gene expression. While for immunofluorescence, fixed larvae can be easily bleached with hydrogen peroxide [199] or other advanced tissue clearing methods [200], the way to transparent in vivo imaging bears hurdles. Phenylthiourea, applied prior to 24 hpf, has been widely used for suppression of pigment development [201,202]. However, it interferes with developmental processes, resulting in malformations due to alterations in retinoic acid, insulin-like growth factor and thyroid hormone signalling [203]. Moreover, it may change xenobiotic metabolism by induction of CYP1A1 enzyme transcription [204] and was shown to alter the toxicity of mercury compounds [205]. Hence, for generating fluorescent lines, transparent zebrafish like *nacre (mitfa^w2^*^/*w2*^*)*, *casper (mitfa^w2^*^/*w2*^; *mpv17^a9^*^/*a9*^*)* or *crystal (mitfa^w2^*^/*w2*^;*alb^b4^*^/*b4*^;*mpv17^a9^*^/*a9*^*)* offer great visualization advantages [206,207]. Alternatives to induce depigmentation in already established transgenic lines without utilizing transparent backgrounds have been explored, for instance deletion of pigment cells via transient CRIPSR/cas9 injections [208].

**Figure 2:**
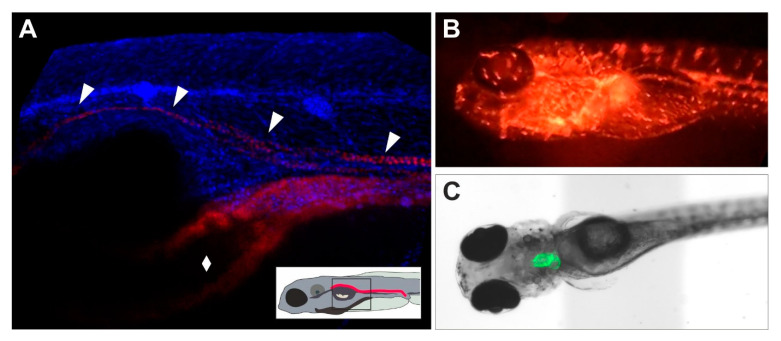
Transgenic zebrafish reporter lines. (**A**) Lateral view of the trunk of a *cdh17:mCherry* larva (5 dpf), co-stained nuclei with Hoechst 33342. The *mCherry* fluorescence labels the kidney (arrowheads) and the intestine (diamond). (**B**) Snapshot of a *gata1:DsRed* larva (5 dpf) in lateral position. Hematopoietic cells are marked by red fluorescence throughout the whole body. (**C**) Ventral *gfp*/brightfield overlay image of the larval *myl7:GFP* heart (5 dpf), exhibiting green fluorescence in myocardial cells around the heart chambers (atrium and ventricle) and in the atrioventricular canal.

Besides their application for morphological assessment of adverse effects in tissues, fluorescent zebrafish lines (Figure 2; Table 2) allow for the isolation of the tagged cells by Fluorescence Activated Cell Sorting (FACS) [209,210,211]. This facilitates isolation of fluorescent labelled organs or target cell populations which, given the small size of the embryos and larvae is hardly possible by any other means [212]. Sorting of the tagged cells enables analysis specifically in target cells, including single-cell gene expression analysis. Aside from continuously labelled organs, transgenic zebrafish lines exist that express fluorescence only under certain conditions, for example in the presence of pollutants in brackish water [213,214,215] or in response to CYP enzyme induction [59]. This principle may provide a valuable approach to biomarker-based assessment of organ toxicity.

### 4.2. Reproducibility and Standardization 

Current toxicity testing in zebrafish is impeded by the lack of experimental protocol standardization and by the resulting lack of reproducibility. One example is the aquatic toxicity analysis of ionizable organic chemicals (IOCs), which requires a well-defined experimental setup, pH and buffer conditions [216]. These critical experimental issues in zebrafish experimentation are increasingly recognized and discussed by the toxicological community [66,67]. One important aspect of this discussion is the current heterogeneity of different breeding conditions in zebrafish research, including the use of varying culture media for embryos (e.g., E3 embryo medium vs. 0.3× Danieau´s medium) [74,217], different well-plate formats for incubation [75,164] and various temperature conditions or illumination status of incubators [54]. Standardization and detailed reporting of breeding conditions of embryos and larvae is crucial for data comparison, reproducibility and reliability. Adult fish also require specific maintenance conditions (e.g., water quality, light/dark cycle, tank size, enrichment, and density) and nutrition (e.g., feeding plan, timing, food composition) for their wellbeing and the generation of viable fry for toxicological experiments, which should be included in reported protocols similar to other vertebrate species (for detailed information see Arrive guidelines: https://arriveguidelines.org/; accessed 9 December 2021). For Europe FELASA (Federation of European Laboratory Animal Science Associations) recommendations have been formulated in greater detail for harmonizing general zebrafish husbandry and health monitoring recommendations [218], which are currently incorporated in local legal regulations in European countries [219]. Further recommendations for zebrafish are worked out at the moment for additional aspects: severity classification in zebrafish and their larvae, methods of humane killing of laboratory fish, pain management in zebrafish, health monitoring of fish in research (https://felasa.eu/working-groups; accessed 9 December 2021). Improved reporting of experimental conditions, detailed protocols and refinement of breeding conditions are indispensable for reproducibility and standardization of future zebrafish toxicological studies.

## 5. Future Perspectives 

Technical advancement has and will have a huge impact on experimentation in zebrafish (Table 3). Several recent developments have opened up far-reaching possibilities for novel applications, reproducibility and standardization in toxicity testing.

### 5.1. Refinement, Automation and High-Throughput Methods

In recent years, a great number every-day processing and handling techniques of zebrafish embryos and larvae have been refined by automation, which normally are a bottleneck in dealing with large animal numbers in semi-/high-throughput toxicological screens. Most times these efforts resulted in a growing number of automated systems for standardized zebrafish handling during experimentation and computer based phenotype quantification, e.g., automated zebrafish egg sorting [220], automated removal of chorions [221], automated imaging systems [222], and automated microinjection [223]. Moreover, a steadily increasing number of follow-up methods for specific topics is being currently developed to further foster toxicological studies in small laboratory fish species. These “custom-tailored” techniques often differ greatly in their experimental setup, costs, measured parameters and scoring values, but show common steps of enhancement such as semi/full-automation of assays [232], streamlining experimental processes [224] and reduction of experimental variances and experimental hands-on time [225]. Examples for these enhancements are automated imaging method advancements, which have been reported for a cystic kidney disease model [226] and for a general automated morphological feature assessment by FISH inspector [227]. Both methods adopted an automatically screening system for zebrafish embryo and larvae handling and subsequently enable uniformly scoring of morphological alterations after toxin application or after genetic alterations.

### 5.2. Advanced Visualization Methods

The fast advancement of imaging techniques and corresponding software in recent times have massively changed the ways how biological samples can be investigated and quantified. Today, image processing, computer aided quantification and feature extraction enable automatic and reproductive measurements, essential for comparative studies in toxicology, e.g., [77]. Most times, these techniques utilize transgenic fluorescent zebrafish lines to mark the organ of interest and subsequently score for a set of distinct parameters (see also Section 3). One recent example is the deep learning-based feature recognition in cardiac function [228,229], enabling reliable and fast quantification of cardiac parameters from high resolution dynamic light-sheet fluorescence or light-field microscopy. The techniques enable visualization and measurement of highly dynamic biological processes, such as heart function, in a living organism in three dimensions over time.

### 5.3. Novel Genetic Methods

Application of advanced genetic methods, like refined next-generation CRIPSR/Cas9 and Next Generation Sequencing (NGS) techniques, have had a huge impact on zebrafish investigations and are likely to continue to do so by enabling establishment of complex transgenic models for rare human disorders [6,233], base pair specific precise genome alterations [230], and by enabling single-cell transcriptome sequencing techniques [234,235,236,237]. These transgenic techniques along with automated microinjection [238] and refined methods of mutation detection [239,240] enable the establishment of “simple” loss-of-function or complex genetic rearrangements. Broad application of transgenic methods become more and more standard and refined protocols allowing to elucidate molecular mechanisms are available even for small labs. In parallel, NGS techniques have been adopted to zebrafish and enable genome wide resequencing for variant identification, expression studies by whole-animal/tissue transcriptome (RNA-seq) or single cell sequencing (scRNA-seq) analyses, successfully implementing Omics-technologies for zebrafish embryos and larvae. Currently, the impact of scRNA-seq on developmental biology of zebrafish is eminent. Although technical demanding, it enables dissection of single cell linages within organs, investigation of stage specific expression changes in single cells and quantitative clustering of cell types [231,235,236,237]. Further examples show how implementation of scRNA-seq either in investigation of zebrafish disease models [241] or in toxicology [242,243] can increase experimental reliability by facilitating high numbers of single cell expression data.

## 6. Conclusions

In summary, the development of novel techniques and refinement of established measurements will have a great impact on how zebrafish embryo and early larval models can be adopted for toxicological screens in the future. Without doubt, this will have a positive impact on the 3Rs, by reducing the number of mammals needed for toxicity testing, by enabling rapid generation of specialized models, by identifying specific compounds in large libraries, or by visualizing adverse effects on organs early during vertebrate development. Complications may arise by the increase in complex data sets and their correct statistical processing, technical demands on the experimental setup, increased experimental costs and detailed reporting of experimental processes. Implementation of advanced, standardized methods might foster cross-species comparative studies to identify fundamental molecular mechanisms and to further establish zebrafish as an alternative or even surrogate toxicological model species to mammals. 

## Figures and Tables

**Table 1:** Comparison of zebrafish toxicological compound studies with focus on cardio-, neuro-, hepato- and nephrotoxicity.

Study	Compounds	Treatment Period	Predictivity	Bioavailability Ensured
Cardiotoxicity				
Alzualde et al., 2015 [68]	Atropine, BAYK8644, Cisapride, Dofetilide, E4031, Flecainide, JNJ303, Quinidine, Salmeterol, Terfenadine, Thiorizidine, Torcetrapib, Verapamil	4 h (48–52 hpf)	Sensitivity 85% Specificity n.a.	Yes
Burns et al., 2005 [69]	Acetaminophen, Allopurinol, Amiodarone, Astemizole, Cimetidine, Tamoxifen	24 h (2–3 dpf)	Sensitivity 100% Specificity 100%	No
Milan et al., 2003 [70]	100 drugs including chlorpromazine, digitoxin and progesterone	4 h at 2 dpf	Sensitivity 96% Specificity 77%	Yes
Zhu et al., 2014 [71]	Aspirin, Clomipramine, Cyclophosphamide monohydrate, Gentamicin sulphate, Nimodipine, Quinidine, Terfenadine, Tetracycline hydrochloride	24 h (2–3 dpf)	Sensitivity 100% Specificity 100%	Yes
**Neurotoxicity**				
Dach et al., 2019 [42]	NTP 91 compound library	up to 114 h (6 hpf–5 dpf)	Sensitivity n.a. Specificity 60%	No
Hagstrom et al., 2019 [72]	NTP 91 compound library	up to 114 h (6 hpf–5 dpf)	Sensitivity 95% Specificity n.a.	No
**Hepatotoxicity**				
Hill et al., 2012 [73]	33 drugs including Troglitazone and Diclofenac	48 h (3–5 dpf)	Sensitivity 91% Specificity 77%	Yes
**Nephrotoxicity**				
Bauer et al., 2021 [74]	Aristolochic acid, Cadmium chloride, Gentamicin, Ochratoxin A, Potassium bromate	48 h (3–5 dpf)	Sensitivity 80% Specificity n.a.	Partially (microinjection of gentamicin)
Gorgulho et al., 2018 [75]	Gentamicin, Paracetamol, Tenofovir, Tenofovir disoproxil fumarate	24 h (4–5 dpf)	Sensitivity 100% Specificity n.a.	No
Westhoff et al., 2013 [76]	Acetaminophen, Ampicillin, Indomethacin, Gentamicin, Kanamycin, Losartan, Penicillin G	24 h (24–48 hpf)	Sensitivity 75% Specificity n.a.	No
Westhoff et al., 2020 [77]	Prestwick chemical library^®^, including 1285 off-patent small molecules, >95% approved drugs	24 h (24–48 hpf)	Sensitivity n.a. Specificity n.a.	No
Wu et al., 2012 [78]	Citrinin, Patulin	42 h (6–48 hpf) 66 h (6–72 hpf) 90 h (6–96 hpf) 24 h (24–48 hpf) 48 h (24–72 hpf) 72 h (24–96 hpf)	Sensitivity 100% Specificity n.a.	No

**Table 2:** Commonly used transgenic lines for cell type-/tissue-specific investigation in zebrafish.

Line (Genomic Feature)	Tagged Structure	Reference *Zfin Line* and *Construct ID*
Cardiovascular system		
*myl7:GFP* *(f1Tg)*	cardiac muscle	[69] *ZDB-ALT-060719-2*; *ZDB-TGCONSTRCT-070117-49*
*gata1:DsRed* *(sd2Tg)*	erythrocytes	[102] *ZDB-ALT-051223-6*; *ZDB-TGCONSTRCT-070117-38*
*cmlc2:gCaMP* *(s878Tg)*	heart specific calcium sensor	[81] *ZDB-ALT-070806-1*; *ZDB-TGCONSTRCT-070806-2*
*fli1:EGFP* *(y1Tg)*	vasculature/blood vessels	[101] *ZDB-ALT-011017-8*; *ZDB-TGCONSTRCT-070117-94*
**Brain and neurological system**		
*mrc1a:eGFP* *(y251Tg)*	glia cells/blood-brain-barrier	[103] *ZDB-ALT-170717-2*; *ZDB-TGCONSTRCT-170717-2*
*elavl3:eGFP* *(knu3Tg)*	general neuronal marker (~HuC)	[104] *ZDB-ALT-060301-2*; *ZDB-TGCONSTRCT-070117-150*
Cre/Lox and Gal4/UAS lines	cell type-specific expression	[105,106] numerous lines and constructs
**Liver**		
*fabp10:eGFP (as3Tg)*	hepatocytes	[107] *ZDB-ALT-060627-2*; *ZDB-TGCONSTRCT-070117-123*
*krt18:eGFP* *(p314Tg)*	biliary cells	[108] *ZDB-ALT-140703-1*; *ZDB-TGCONSTRCT-140703-1*
*hand2:eGFP* *(pd24Tg)*	stellate cells	[109] *ZDB-ALT-110128-40*; *ZDB-TGCONSTRCT-110128-8*
**Kidney**		
*wt1b:GFP* *(li1Tg)*	glomerulus, proximal tubule	[110] *ZDB-ALT-071127-1*; *ZDB-TGCONSTRCT-071127-1*
*PT:eGFP* *(nz4Tg)*	proximal tubule	[111] *ZDB-ALT-150414-3*; *ZDB-TGCONSTRCT-150414-3*
*cdh17:eGFP* *(zf237Tg)*	proximal and distal tubule	[112] *ZDB-ALT-110525-2*; *ZDB-TGCONSTRCT-110525-1*
*enpep:eGFP* *(p152Tg)*	proximal and distal tubule	[113] *ZDB-ALT-101123-3*; *ZDB-TGCONSTRCT-101123-2*
*pax8:mCherry* *(nia03Gt)*	distal tubule	[114] *ZDB-ALT-110711-15*; *ZDB-GTCONSTRCT-110322-1*
*pod:mCherry* *(zf238Tg)*	glomerulus	[112] *ZDB-ALT-110525-3*; *ZDB-TGCONSTRCT-110525-2*

**Table 3:** Novel techniques enhancing toxicological investigation in zebrafish.

Technique	Advantage for Toxicology	References
Refinement, Automation and High-Throughput Methods		
Automated zebrafish egg sorting	less hands-on time	[220]
Automated removal of chorions	less hands-on time, prerequisite for toxicological screens	[221]
Automated imaging systems	standardization of imaging and visual screening	[222]
Automated microinjection	standardization of microinjection	[223]
Dechorionated Zebrafish Embryo Developmental toxicity assay or culture assay	harmonized zebrafish developmental toxicology assay to assess teratogenic liability of pharmaceutical compounds	[224,225]
Cystic kidney disease model	automated morphological feature assessment	[226]
FISH inspector	automated morphological feature assessment	[227]
Multiparametric renal function assay	assessment of pronephric morphology, renal function and heart Rate	[186]
**Advanced visualization methods**		
Computer aided automation in imaging analyses	high throughput method for imaging data, highly comparable, standardised results	[77]
Automatic feature recognition	automatic quantification of changes, comparable, predefined parameters	[228,229]
**Novel genetic methods**		
Next-gen CRISPR/Cas9, single nucleotide editing	establishment of precise genetic modifications	[230]
RNA-seq	whole-animal/tissue transcriptome analyses	Gene expression atlas for zebrafish developmental stages: http://www.ebi.ac.uk/gxa/experiments/E-ERAD-475; accessed on 9 December 2021
scRNA-seq	single cell transcriptome analyses	[231]

## Data Availability

Not applicable.
